# Oropharyngeal Candidiasis in HIV Infection: Analysis of Impaired Mucosal Immune Response to *Candida albicans* in Mice Expressing the HIV-1 Transgene

**DOI:** 10.3390/pathogens4020406

**Published:** 2015-06-23

**Authors:** Louis de Repentigny, Mathieu Goupil, Paul Jolicoeur

**Affiliations:** 1 Department of Microbiology, Infectious Diseases and Immunology, Faculty of Medicine, University of Montreal, C.P. 6128, succursale Centre-Ville, Montreal, PQ H3C 3J7, Canada; E-Mail: mathieu.goupil@umontreal.ca; 2 Laboratory of Molecular Biology, Clinical Research Institute of Montreal, 110, avenue des Pins Ouest, Montreal, PQ H2W 1R7, Canada; E-Mail: paul.jolicoeur@ircm.qc.ca

**Keywords:** Candidiasis, human immunodeficiency virus (HIV), AIDS, mucosal immunity, transgenic mice

## Abstract

IL-17-producing Th17 cells are of critical importance in host defense against oropharyngeal candidiasis (OPC). Speculation about defective Th17 responses to oral *C. albicans* infection in the context of HIV infection prompted an investigation of innate and adaptive immune responses to *Candida albicans* in transgenic mice expressing the genome of HIV-1 in immune cells and displaying an AIDS-like disease. Defective IL-17 and IL-22-dependent mucosal responses to *C. albicans* were found to determine susceptibility to OPC in these transgenic mice. Innate phagocytes were quantitatively and functionally intact, and individually dispensable for control of OPC and to prevent systemic dissemination of *Candida* to deep organs. CD8+ T-cells recruited to the oral mucosa of the transgenic mice limited the proliferation of *C. albicans* in these conditions of CD4+ T-cell deficiency. Therefore, the immunopathogenesis of OPC in the context of HIV infection involves defective T-cell-mediated immunity, failure of crosstalk with innate mucosal immune effector mechanisms, and compensatory cell responses, which limit *Candida* infection to the oral mucosa and prevent systemic dissemination.

## 1. Introduction

Susceptibility to oropharyngeal candidiasis (OPC) in patients living with HIV/AIDS has prompted intense investigation of the mechanisms causing defective mucosal immunity to *Candida albicans* in this clinical setting. Surveys conducted since the onset of the HIV/AIDS pandemic have consistently shown that OPC is closely correlated to reduced CD4+ T-cell counts in peripheral blood [1,2,3,4,5]. The virtual absence of CD4+ cells in the oral mucosa of HIV-infected patients with the erythematous or pseudomembranous forms of OPC [6,7,8], as well as lower tissue densities of this cell population in HIV-positive compared to HIV-negative controls without OPC [6], further suggested a role for CD4+ cell depletion in the immunopathogenesis of OPC in the setting of HIV/AIDS. During the period of the Th1/Th2 paradigm (1986–2005) [9], susceptibility to OPC in HIV infection was attributed to a defective Th1 response to *C. albicans* and/or shift to a non-protective Th2 response [10,11], reviewed in [12]. Nevertheless, many questions remained unanswered, particularly the identity of the CD4+ cell-dependent downstream effector mechanism(s) whose loss triggers transition of the *C. albicans*-host interaction from commensalism to infection, and the crosstalk between these effector mechanisms and *Candida*-specific CD4+ T-cells. The discovery of IL-17-producing CD4+ T-cells in 2005, extending the existing Th1/Th2 paradigm to include a distinct effector Th17 cell lineage [13], prompted a reassessment and novel understanding of the mechanisms of host defense against *C. albicans* in the normal host [14,15] and, by extension, of the defects of these mechanisms in the setting of HIV infection.

## 2. IL-17- and IL-22-Dependent Mucosal Host Response to *Candida albicans*

**Figure 1:**
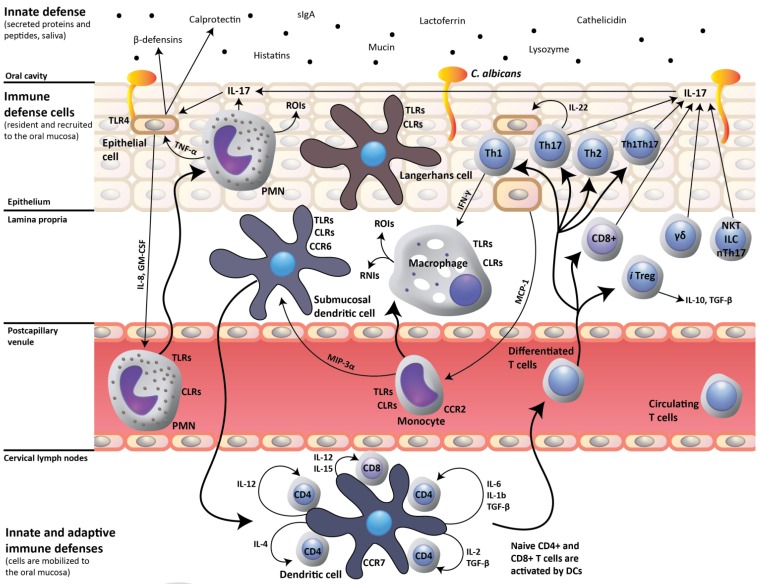
Host response to oral *Candida albicans*. A protective host response to oral *C. albicans* infection is dependent on dendritic cell-mediated induction of Th17 cell-mediated adaptive immunity, which, by the production of IL-17 upregulates the innate expression of mucosal antimicrobial peptides (β-defensins, calprotectin) by epithelial cells. IL-17 also up-regulates IL-8 and GM-CSF production by epithelial cells, which in turn trigger recruitment of neutrophils to the oral mucosa. Innate-like cell populations, including γδ T-cells, NKT cells, ILCs and nTh17 cells, also produce IL-17 and may participate in the mucosal host response. CLRs, C-type lectin receptors; RNIs, reactive nitrogen intermediates; ROIs, reactive oxygen intermediates; and TLRs, toll-like receptors.

In a landmark investigation, Conti *et al.*, [16] addressed inconsistencies in the prevailing view that the Th1 CD4+ T-cell subset is chiefly responsible for oral mucosal host defense against *C. albicans*. Previous investigation had shown that IL-12p40-knockout mice are deficient in Th1 cells and susceptible to OPC, but that IFN-γ-knockout mice also have a defective Th1 response and are resistant to OPC [17]. This discrepancy was resolved when it was realized that IL-12p40 is common to the dimeric cytokines IL-12 and IL-23, also composed of the specific components IL-12p35 and IL-23p19, respectively. Conti *et al.* elegantly demonstrated that IL-23p19-knockout mice are susceptible to OPC and display impaired neutrophil recruitment to the mucosa, while IL-12p35 mice are relatively resistant [16]. They also reported that Th17 signature genes are induced early after oral *C. albicans* infection of immunocompetent mice, and that mucosal expression of murine β-defensin 3, S100A8 and CCL20 is defective in IL-17RA-knockout mice [16]. These results conclusively showed that IL-17 produced by Th17 cells, and not IL-12 produced by Th1 cells, is critical to a protective host response against OPC, and that neutrophils and mucosal antimicrobial peptides are the primary downstream effector mechanisms of the Th17 anticandidal immune response (Figure 1). In addition to IL-17, IL-22 production by Th17 cells was also shown to contribute to early host protection against *C.* *albicans* [16,18,19], which concurred with *in vitro* evidence that IL-17 and IL-22 cooperatively enhance expression of antimicrobial peptides by oral keratinocytes [20,21,22,23,24] (Figure 1).

Since the publication of the Conti study [16], IL-17-producing cell populations other than conventional Th17 cells, including γδ T-cells [25], NKT cells [26], Tc17 CD8+ T-cells [27], innate lymphoid cells (ILCs) [28], and natural Th17 (nTh17) cells [29] have been the subject of intense interest, considering that they may also participate in the protective mucosal response to *C. albicans* [16,30,31,32]. Of direct relevance to OPC in HIV infection, Tc17 cells isolated from re-challenged CD4+ knockout mice serve as an alternate source of IL-17 after transfer into Rag-knockout recipients, conferring protection against OPC [31]. Therefore, in conditions of CD4-deficiency, Tc17 CD8+ T-cells are able to promote effective immunity to *C. albicans* [31]. Nevertheless, a challenging question remained: how is it possible that Th17 cells conferred protection against OPC as early as three days after oral infection with *C. albicans* [16], considering that a far longer period is required for the induction of an adaptive cell-mediated immune response? Of the “innate-like” IL-17-producing cell populations that could potentially be involved in early protection after infection, γδ T-cells and NKT cells have been shown to not contribute significantly to IL-17-mediated immunity to *C. albicans* in the oral mucosa [16,30]. However, a protective oral mucosal response to *C. albicans* was shown to be mediated by IL-17-producing ILCs and not by Th17 cells [30]. Group 3 ILCs, including Lymphoid Tissue inducer cells (LTi), are under the control of the RORγt transcription factor and produce IL-17 and/or IL-22 [33]. More recently, a role for ILCs was questioned when it was found that IL-17 is produced within 24–48 h by tongue-resident nTh17 cells, but not by ILCs [29]. Although further work remains to be done to resolve these different findings, it is now clear that several redundant IL-17-producing cell populations are involved in the protective response to oral *C. albicans* infection in experimentally infected mice. Nevertheless, it must be kept in mind that the limited time course of experimental primary oral infection in mice, a species in which *C. albicans* is not a pre-existing commensal, is far different from the lifelong commensalism and adaptive immunity to *C. albicans* established shortly after birth in humans. Accordingly, further studies are needed to delineate the relative contributions of individual IL-17-producing cell populations that maintain the state of *C. albicans* commensalism in healthy humans, and to uncover alterations in IL-17-mediated protective mechanisms which cause susceptibility to OPC in specific primary or acquired immunodeficiencies. As a first step towards this goal, the critical role of IL-17-mediated mucosal immunity to *C. albicans* in humans was established in patients with chronic mucocutaneous candidiasis, with five genetic etiologies: (i) autosomal recessive deficiency in the IL-17 receptor A [34], (ii) autosomal dominant deficiency of IL-17F [34], (iii) autosomal dominant gain of *STAT1* activity [35], (iv) mutation of *ACT1* [36], or (v) autosomal recessive IL-17RC deficiency [37]. Other studies identified individuals with a mutated form of the C-type lectin receptor dectin-1 that leads to defective production of IL-6 and IL-17 and mucocutaneous candidiasis [38], and patients with Job’s syndrome (hyper-IgE syndrome) in whom mutations in *STAT3* cause a lack of antigen-specific Th17 cells and increased susceptibility to mucocutaneous infections with *C. albicans* [39]. Taken together, these human studies showed that several primary immune deficiencies share a defective IL-17-mediated immune response to *C. albicans* as the common cause of susceptibility to OPC, but each perturbing distinct steps in the generation of the IL-17-mediated response (reviewed in [40]).

## 3. Impact of HIV Infection on Oral Mucosal Immunity to *C. albicans*

Human HIV infection profoundly alters oral mucosal immune cell populations, which have been implicated in host defense against *C. albicans.* The impact of HIV infection on dendritic cells and CD4+ T-cells, which both harbour HIV, is most striking.

During sexual transmission of HIV, mucosal dendritic cells (DCs) play a critical role in HIV capture and transfer to CD4+ T-cells, and are therefore crucial in the establishment of primary (acute) HIV infection. DCs also participate in the pathological immune activation observed in chronic HIV infection (reviewed in [41]). HIV-infected patients display multiple defects of the oral Langerhans cell population of DCs. Both oral [42] and esophageal [43] Langerhans cells are depleted in HIV infection, congruent with decreased numbers of cervical [44], splenic [45] and blood [46,47,48,49,50] DCs. In addition, early studies revealed impairment of terminal differentiation of oral Langerhans cells, evidenced by decreased expression of MHC class II antigens [6,51], and the presence of blunt dendrites, very limited development of organelles, and lack of Birbeck granules [6]. HIV-1 nef, acting via activation of PAK2, inhibits DC maturation [52] as shown by reduced expression of MHC class I, CD80 and CD86 [53], and impairs DC antigen presentation to T-cells [53,54]. The nef protein also down-modulates surface expression of MHC class II [55,56], induces the intracellular accumulation of MHC class II–peptide complexes [56], and uncouples cytokine and chemokine production from membrane phenotype maturation in DCs [57]. Nef also reduces mannose receptor expression on DCs, which may further cripple the host innate immune response [58]. These findings indicate that defective DCs could potentially contribute to a loss of protective CD4+ T-cell-mediated immune responses to *C. albicans* in HIV infection, by their impaired presentation of *C. albicans* antigens and altered production of cytokines required for CD4+ T-cell subset differentiation. Congruent with this hypothesis, inflammatory DCs from normal humans have been shown to induce Th17 cell differentiation [59]. However, a role for defective DCs in susceptibility to OPC may not exist in adult humans with HIV/AIDS, because *Candida* antigens have already been presented by intact DCs to CD4+ T-cells in infancy, thereby successfully generating *Candida*-specific memory CD4+ T-cells [60] many years before the acquisition of HIV. Accordingly, impairments of DCs resulting from subsequent HIV infection in adulthood would be predicted to not contribute to the loss of existing *Candida*-specific memory CD4+ cells, unless continued low-level presentation of *Candida* antigens and/or production of cytokines by DCs is required throughout life to maintain this cell population. However, it is now clear that memory CD4+ T-cells are maintained through contact with IL-7 and IL-15, but not from TCR interaction with MHC class II ligands [61,62]. Accordingly, there is at present no compelling evidence to support the notion that HIV-induced defects of DCs would contribute to susceptibility to OPC in human HIV infection. This conclusion stands in contrast to the situation in mice experimentally infected with *C. albicans*, which undergo a primary infection with *Candida* and generation of a Th17 response requiring cytokine production and antigen presentation by DCs [63]. Therefore, in mice, defective DCs could potentially contribute to a defective Th17 response to *C. albicans* and susceptibility to OPC.

In normal humans, memory CD4+ T-cells specific for *C. albicans* reside mainly in the Th17 subset [64,65]. It has been clearly established that CCR6+ Th17 cells, including those specific to *C. albicans*, are highly permissive to HIV-1 *in vitro* and are preferentially depleted in peripheral blood of patients with HIV/AIDS [66,67,68,69,70,71]. Evidence has also been presented showing that Th17 cells are depleted in the gastrointestinal mucosa of persons infected with HIV [72,73,74]. A survey of the scientific literature reveals sustained speculation about potentially defective Th17 responses to oral *C. albicans* in the context of human HIV infection [14,40,75,76], which would result in a lack of the critical cytokines IL-17 and IL-22 required to up-regulate the innate mucosal response, and consequently cause susceptibility to OPC [77]. However, formal cause-and-effect between Th17 cell depletion and susceptibility to OPC in patients with HIV/AIDS has been lacking, and would likely be extremely difficult to establish because of ethical considerations and the confounding effects of antiretroviral and antifungal therapies [78]. For these reasons, we have employed a model of oral *Candida* infection in transgenic (Tg.) mice expressing HIV-1 to elucidate the mechanisms of impaired mucosal immunity, which cause susceptibility to OPC.

## 4. Oral *C. albicans* Infection in CD4C/HIV Transgenic Mice: Evidence for Defective IL-17- and IL-22-Mediated Mucosal Host Response

CD4C/HIV Tg mice were initially developed as a tool to study the immunopathogenesis of HIV/AIDS in a small laboratory animal, with the aim of reproducing as faithfully as possible the pathological and immune perturbations observed in human HIV infection [79]. These Tg mice express selected HIV-1 genes, under control of CD4C regulatory elements, in thymic immature double-positive CD4+ CD8+ and mature CD4+ CD8- T-cells, peripheral mature CD4+ T-cells, macrophages and DCs, which comprise the main immune cell populations infected with HIV-1 in humans ([79], reviewed in [12]). Selective expression of the HIV-1 Nef gene in these Tg mice is necessary and sufficient to induce an AIDS-like disease [79], characterized by failure to thrive, wasting, severe atrophy and fibrosis of lymphoid organs, a preferential depletion of CD4+ T-cells, with altered CD4+ T-cell proliferation *in vitro*, loss of CD4+ T-cell help, CD4+ T-cell and B-cell activation and impaired DC maturation and function [54,79,80,81,82,83]. Congruent with human HIV infection, transgenic DCs show a decreased capacity to present antigen *in vitro,* consistent with their reduced MHC class II expression and impaired maturation profile [31]. In addition, disease of the lung (lymphocytic interstitial pneumonitis), heart (myocytolysis, myocarditis), and kidney (segmental glomerulosclerosis, tubulointerstitial nephritis, microcystic dilatation) develop in these Tg mice [79,84].

CD4C/HIV^MutA^ Tg mice, which express the Nef, Env and Rev genes of HIV-1, fail to clear *C. albicans* from their oral cavities after intra-oral inoculation, and maintain chronic oral carriage of *C. albicans* over several months, in striking contrast to non-Tg littermates, which clear the fungus within seven days following inoculation [85]. Congruent with human HIV infection, *Candida* hyphae penetrate the superficial layer of the oral epithelium of chronically infected Tg mice, with a mononuclear cell infiltrate, and dissemination to deep organs is limited [85]. Subsequent investigation showed that quantitative and functional defects of both DCs and CD4+ T-cells determine susceptibility to OPC in these Tg mice [86], evidenced by (i) depletion of DCs, which show an immature phenotype; (ii) depletion of CD4+ T-cells, which fail to proliferate and to acquire an effector phenotype in response to *C. albicans* antigen *in vitro*; (iii) reduced proliferation of CD4+ T-cells and their production of IL-2, on coculture of *C.* *albicans*-pulsed DCs with CD4+ T-cells *in vitro*, each cell population expressing or not the HIV-1 transgene; and (iv) restored proliferation to *C. albicans* antigen and sharply reduced oral burdens of *C. albicans*, after transfer of CD4+ T-cells from uninfected non-Tg mice into infected Tg mice [86]. Further work showed that restricted HIV-1 Nef expression in DCs and macrophages, combined or not with expression in CD4+ T-cells, leads to persistent oral carriage of *C. albicans* [87], confirming that the phenotype of susceptibility to OPC in this transgenic mouse model results from perturbations of both DCs and CD4+ T-cells.

**Figure 2:**
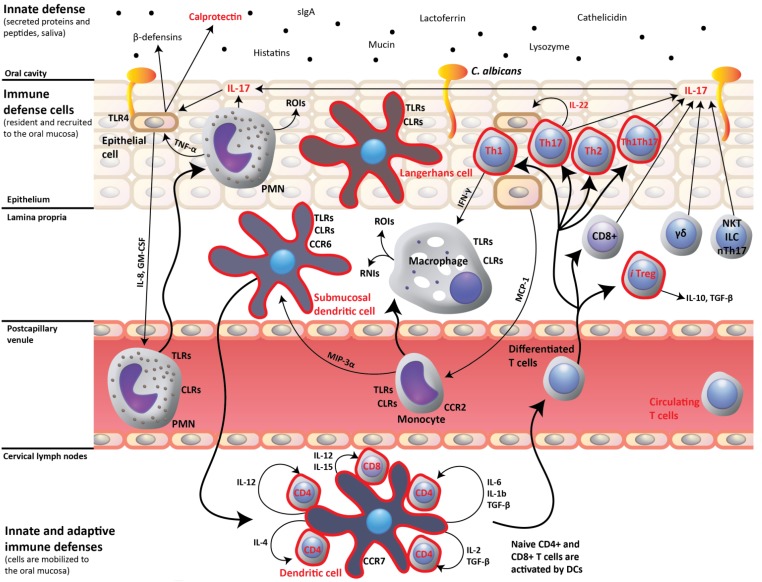
The protective Th17 cell-mediated mucosal immune response to *Candida albicans* is perturbed (red color) in CD4C/HIV Mut transgenic mice. Dendritic cells display an immature phenotype and defective antigen presentation. Naïve CD4+ T cells, and differentiated CD4+ T-cell subsets, including Th17 cells, are all depleted in these transgenic mice, which fail to up-regulate oral mucosal expression of *IL17*, *IL22* and *S100a8* in response to oral *C. albicans* infection. CLRs, C-type lectin receptors; RNIs, reactive nitrogen intermediates; ROIs, reactive oxygen intermediates; and TLRs, toll-like receptors.

Demonstration of the critical role of Th17 cells in mucosal protection against OPC [16] led us to analyse CD4+ T-cell subsets and to seek direct evidence for a defective IL-17-dependent response to oral *C. albicans* in the CD4C/HIV^MutA^ Tg mice [88] (Figure 2). As expected, naïve CD4+ T-cells and the differentiated Th1, Th2, Th17, Th1Th17 and Treg lineages were all profoundly depleted in cervical lymph nodes of the Tg mice. To determine if this subset depletion is caused by a lack of naïve cells and/or inability of naïve cells to respond to differentiating cytokines, identical numbers of naïve cells from Tg and non-Tg mice were incubated *in vitro* with cocktails of differentiating cytokines specific to each CD4+ T-cell subset [88]. It turned out that naïve CD4+ T-cells from Tg mice maintained the capacity to differentiate into the specific lineages in response to polarizing cytokines and to produce the critical cytokines, including IL-17A in the case of Th17 differentiating conditions, required for a protective adaptive immune response to *C. albicans*. These findings indicated that depletion of polarized CD4+ T-cell subsets in the Tg mice is most likely caused by the marked diminution of naïve CD4+ cells, rather than any potential downstream defects in CD4+ cell differentiation [88].We next showed that oral infection with *C. albicans* induced expression of *S100a8*, *Ccl20*, *Il17* and *Il22* in tongue tissues of non-Tg mice, but that this mucosal immune response to *C. albicans* infection was completely abrogated in the Tg mice. Furthermore, treatment of infected Tg mice with the combination of IL-17 and IL-22 significantly reduced oral burdens of *C. albicans*, markedly decreased the density of *C. albicans* on histopathology of the oral epithelium, and restored the expression of *S100a8* and *Ccl20* [88]. Therefore, in the context of HIV-expression, cause-and-effect was established between defective IL-17 and IL-22 responses, perturbed antimicrobial peptide gene expression, and susceptibility to oral *C. albicans* infection. Interestingly, Il-17 and Il-22 alone did not significantly reduce oral loads of *C. albicans*, showing that neither cytokine is dispensable for protection against OPC in this animal model [88]. Furthermore, untreated Tg mice displayed a progressive reduction in oral burdens of *C. albicans* from day 5 to 17 after oral infection, suggesting the participation of IL-17-producing cell populations other than Th17 cells in the response to OPC in the Tg mice, which could potentially include γδ T-cells, NKT cells, Tc17 CD8 T-cells, and ILCs.

No discernable influx of neutrophils was induced by the cytokine treatment. This was expected on the part of IL-22, which does not act on immune cells [21] and is uninvolved in neutrophil recruitment to the oral mucosa in murine candidiasis [16]. However, we were initially surprised to find that IL-17 treatment of infected Tg mice failed to produce a neutrophil influx at day 7 after infection, considering that neutrophil recruitment in murine OPC had been previously shown to be largely IL-17-dependent [16,89]. To explain this apparent inconsistency, it is important to recognize that these previous observations were done five days after primary oral infection of immunocompetent mice with *C. albicans* [16]. Although we have not as yet examined neutrophil recruitment in the Tg mice at time points earlier than seven days after infection, early [90] and more recent [91] studies of experimental OPC in immunocompetent mice have consistently shown that the early neutrophil influx is maximal at 24–72 h after infection with *C. albicans* and is largely replaced by mononuclear cells after day 7 of infection. A predominantly mononuclear cell response to *C. albicans* is also observed in patients with the pseudomembranous form of OPC [92], and in CD4C/HIV^MutA^ Tg mice with chronic carriage of *C. albicans*, examined at a time point of several months after intra-oral inoculation and primary infection [85]. Furthermore, although the Tg mice display increased numbers of circulating neutrophils, depletion of these cells beginning on day 20, 45 or 63 after oral inoculation does not alter oral burdens of *C. albicans* or cause systemic dissemination [93]. Therefore, neutrophils do not appear to contribute, at least non-redundantly, to host protection against chronically established OPC. 

Taken together, these findings demonstrate that susceptibility to OPC in HIV-transgenic mice is caused by a defective IL-17- and IL-22-mediated adaptive immune response to *C. albicans*, which in turn causes a failure to up-regulate innate antimicrobial peptide-mediated protective immunity in the oral mucosa. Nevertheless, because HIV transgene expression begins at the onset of life in the Tg mice, and in all CD4+ cells as compared to a low percentage in human HIV infection, and is only later followed by *C. albicans* infection, the direct applicability of these findings to HIV-infected humans remains to be determined.

## 5. Oral *C. albicans* Infection in CD4C/HIV Transgenic Mice: Evidence for Protective CD8+ T-Cell Response

Early histopathological examination of oral biopsies from HIV-infected patients with OPC [6,7], and oral tissues from chronically infected CD4C/HIV^MutA^ Tg mice [85], revealed that *Candida* hyphae invade the superficial layer of the epithelium but do not penetrate beyond the spinous cell layer. This key observation, combined with the low incidence of *Candida* dissemination to deep organs, suggested that some residual mucosal immunity, mediated either by the remaining CD4+ T-cells, or by neutrophils, macrophages or CD8+ T-cells, can compensate in part for the CD4+ T-cell defect caused by HIV infection. 

Analysis of the oral macrophage response to *C. albicans* in CD4C/HIV^MutA^ Tg mice revealed that no quantitative or functional macrophage defect directly causes susceptibility to mucosal candidiasis in these Tg mice [94]. Macrophages from the Tg mice display an alternatively activated phenotype (M2), and production of NO by macrophages is not required to limit mucosal proliferation of *C. albicans* or to prevent systemic dissemination. Likewise, neutrophils from Tg mice, isolated at >90% purity from a double density gradient, were found to be quantitatively and functionally unimpaired, and also not required for control of mucosal and systemic candidiasis in these Tg mice [93]. Therefore, macrophages and neutrophils are both intact and individually dispensable during chronic carriage of *C. albicans* in this T-cell-defective host. Nevertheless, the potential redundancy of these two cell populations remains to be investigated.

Studies in HIV-infected patients with OPC [8,11,95] and in CD4C/HIV^MutA^ Tg mice [86] have shown an influx of CD8+ T-cells to the oral mucosa in response to *C. albicans* infection, suggesting that this cell population may play a role in limiting oral proliferation in the context of CD4+ T-cell deficiency. This hypothesis was confirmed by elevated oral burdens of *C. albicans* in CD4C/HIV^MutG^ CD8^−/−^ compared to CD4C/HIV^MutG^ Tg mice, throughout the chronic carrier phase of infection [93]. Augmentation of oral burdens of *C. albicans* in CD8^−/−^ mice occurred in animals that also express the CD4C/HIV^MutG^ transgene, but not in control mice, which do not express the transgene [93]. Further, absence of CD8+ T-cells in the CD4C/HIV^MutG^ Tg mice did not enhance systemic dissemination of *C. albicans* [93]. These results demonstrated for the first time that CD8+ T-cells participate in host defense against OPC *in vivo,* specifically in the context of HIV Nef expression in a subset of immune cells [93]. It will be relevant to further characterize the CD8+ T-cells recruited to the oral mucosa of Tg mice infected with *C. albicans*, to determine if they belong to the IL-17-producing Tc17 CD8+ T-cell subset [27].

## 6. Conclusion

Studies conducted in experimentally infected mice and in patients with primary immune deficiencies have established that IL-17 produced by Th17 cells is critical to a protective host response against OPC. Using a model of OPC in transgenic mice selectively expressing the genome of HIV-1 and displaying an AIDS-like disease, we have demonstrated that defective IL-17- and IL-22-dependent induction of innate mucosal immunity to *C. albicans* is central to the phenotype of susceptibility to OPC. Therefore, an intact crosstalk between adaptive and innate mucosal immunity is of critical importance to maintain *Candida* in a state of commensalism and to prevent the onset of infection. The discovery of Th22 cells as a distinct human CD4+ T-cell subset [96] and the demonstration of phenotypic plasticity of Th17 cells in lymphopenic hosts [97] both illustrate the need for further investigation of the mechanisms of defective mucosal immunity to *C. albicans* in HIV infection.

## References

[1] McCarthy G.M. (1992). Host factors associated with HIV-related oral candidiasis. A review. Oral Surg. Oral Med. Oral Pathol..

[2] McCarthy G.M., Mackie I.D., Koval J., Sandhu H.S., Daley T.D. (1991). Factors associated with increased frequency of HIV-related oral candidiasis. J. Oral Pathol. Med..

[3] Nielsen H., Bentsen K.D., Hojtved L., Willemoes E.H., Scheutz F., Schiodt M., Stoltze K., Pindborg J.J. (1994). Oral candidiasis and immune status of HIV-infected patients. J. Oral Pathol. Med..

[4] Mercante D.E., Leigh J.E., Lilly E.A., McNulty K., Fidel P.L. (2006). Assessment of the association between HIV viral load and CD4 cell count on the occurrence of oropharyngeal candidiasis in HIV-infected patients. J. Acquir. Immune Defic. Syndr..

[5] Campo J., Del Romero J., Castilla J., Garcia S., Rodriguez C., Bascones A. (2002). Oral candidiasis as a clinical marker related to viral load, CD4 lymphocyte count and CD4 lymphocyte percentage in HIV-infected patients. J. Oral Pathol. Med..

[6] Romagnoli P., Pimpinelli N., Mori M., Reichart P.A., Eversole L.R., Ficarra G. (1997). Immunocompetent cells in oral candidiasis of HIV-infected patients: An immunohistochemical and electron microscopical study. Oral Dis..

[7] Reichart P.A., Philipsen H.P., Schmidt-Westhausen A., Samaranayake L.P. (1995). Pseudomembranous oral candidiasis in HIV infection: Ultrastructural findings. J. Oral Pathol. Med..

[8] Myers T.A., Leigh J.E., Arribas A.R., Hager S., Clark R., Lilly E., Fidel P.L. (2003). Immunohistochemical evaluation of T cells in oral lesions from human immunodeficiency virus-positive persons with oropharyngeal candidiasis. Infect. Immun..

[9] Mosmann T.R., Cherwinski H., Bond M.W., Giedlin M.A., Coffman R.L. (2005). Two types of murine helper T cell clone. I. Definition according to profiles of lymphokine activities and secreted proteins. 1986. J. Immunol..

[10] Fidel P.L. (2011). Candida-host interactions in HIV disease: Implications for oropharyngeal candidiasis. Adv. Dent. Res..

[11] Fidel P.L. (2006). Candida-host interactions in HIV disease: Relationships in oropharyngeal candidiasis. Adv. Dent. Res..

[12] De Repentigny L., Lewandowski D., Jolicoeur P. (2004). Immunopathogenesis of oropharyngeal candidiasis in human immunodeficiency virus infection. Clin. Microbiol. Rev..

[13] Harrington L.E., Hatton R.D., Mangan P.R., Turner H., Murphy T.L., Murphy K.M., Weaver C.T. (2005). Interleukin 17-producing CD4+ effector T cells develop via a lineage distinct from the T helper type 1 and 2 lineages. Nat. Immunol..

[14] Conti H.R., Gaffen S.L. (2010). Host responses to Candida albicans: Th17 cells and mucosal candidiasis. Microbes Infect..

[15] Pirofski L.A., Casadevall A. (2009). Rethinking T cell immunity in oropharyngeal candidiasis. J. Exp. Med..

[16] Conti H.R., Shen F., Nayyar N., Stocum E., Sun J.N., Lindemann M.J., Ho A.W., Hai J.H., Yu J.J., Jung J.W. (2009). Th17 cells and IL-17 receptor signaling are essential for mucosal host defense against oral candidiasis. J. Exp. Med..

[17] Farah C.S., Hu Y., Riminton S., Ashman R.B. (2006). Distinct roles for interleukin-12p40 and tumour necrosis factor in resistance to oral candidiasis defined by gene-targeting. Oral Microbiol. Immunol..

[18] De Luca A., Zelante T., D’Angelo C., Zagarella S., Fallarino F., Spreca A., Iannitti R.G., Bonifazi P., Renauld J.C., Bistoni F. (2010). IL-22 defines a novel immune pathway of antifungal resistance. Mucosal Immunol..

[19] Eyerich S., Wagener J., Wenzel V., Scarponi C., Pennino D., Albanesi C., Schaller M., Behrendt H., Ring J., Schmidt-Weber C.B. (2011). IL-22 and TNF-alpha represent a key cytokine combination for epidermal integrity during infection with Candida albicans. Eur. J. Immunol..

[20] Liang S.C., Tan X.Y., Luxenberg D.P., Karim R., Dunussi-Joannopoulos K., Collins M., Fouser L.A. (2006). Interleukin (IL)-22 and IL-17 are coexpressed by Th17 cells and cooperatively enhance expression of antimicrobial peptides. J. Exp. Med..

[21] Wolk K., Witte E., Witte K., Warszawska K., Sabat R. (2010). Biology of interleukin-22. Semin. Immunopathol..

[22] Wolk K., Kunz S., Witte E., Friedrich M., Asadullah K., Sabat R. (2004). IL-22 increases the innate immunity of tissues. Immunity.

[23] Kolls J.K., McCray P.B., Chan Y.R. (2008). Cytokine-mediated regulation of antimicrobial proteins. Nat. Rev. Immunol..

[24] Peck A., Mellins E.D. (2010). Precarious balance: Th17 cells in host defense. Infect. Immun..

[25] O’Brien R.L., Roark C.L., Born W.K. (2009). IL-17-producing gammadelta T cells. Eur. J. Immunol..

[26] Rachitskaya A.V., Hansen A.M., Horai R., Li Z., Villasmil R., Luger D., Nussenblatt R.B., Caspi R.R. (2008). Cutting edge: NKT cells constitutively express IL-23 receptor and RORgammat and rapidly produce IL-17 upon receptor ligation in an IL-6-independent fashion. J. Immunol..

[27] Hamada H., Garcia-Hernandez Mde L., Reome J.B., Misra S.K., Strutt T.M., McKinstry K.K., Cooper A.M., Swain S.L., Dutton R.W. (2009). Tc17, a unique subset of CD8 T cells that can protect against lethal influenza challenge. J. Immunol..

[28] Walker J.A., Barlow J.L., McKenzie A.N. (2013). Innate lymphoid cells--how did we miss them?. Nat. Rev. Immunol..

[29] Conti H.R., Peterson A.C., Brane L., Huppler A.R., Hernandez-Santos N., Whibley N., Garg A.V., Simpson-Abelson M.R., Gibson G.A., Mamo A.J. (2014). Oral-resident natural Th17 cells and gammadelta T cells control opportunistic Candida albicans infections. J. Exp. Med..

[30] Gladiator A., Wangler N., Trautwein-Weidner K., LeibundGut-Landmann S. (2013). Cutting edge: IL-17-secreting innate lymphoid cells are essential for host defense against fungal infection. J. Immunol..

[31] Hernandez-Santos N., Huppler A.R., Peterson A.C., Khader S.A., McKenna K.C., Gaffen S.L. (2013). Th17 cells confer long-term adaptive immunity to oral mucosal Candida albicans infections. Mucosal. Immunol..

[32] Gladiator A., LeibundGut-Landmann S. (2013). Innate lymphoid cells: New players in IL-17-mediated antifungal immunity. PLoS Pathog..

[33] Luci C., Reynders A., Ivanov II, Cognet C., Chiche L., Chasson L., Hardwigsen J., Anguiano E., Banchereau J., Chaussabel D. (2009). Influence of the transcription factor RORgammat on the development of NKp46+ cell populations in gut and skin. Nat. Immunol..

[34] Puel A., Cypowyj S., Bustamante J., Wright J.F., Liu L., Lim H.K., Migaud M., Israel L., Chrabieh M., Audry M. (2011). Chronic mucocutaneous candidiasis in humans with inborn errors of interleukin-17 immunity. Science.

[35] Boisson-Dupuis S., Kong X.F., Okada S., Cypowyj S., Puel A., Abel L., Casanova J.L. (2012). Inborn errors of human STAT1: Allelic heterogeneity governs the diversity of immunological and infectious phenotypes. Curr. Opin. Immunol..

[36] Boisson B., Wang C., Pedergnana V., Wu L., Cypowyj S., Rybojad M., Belkadi A., Picard C., Abel L., Fieschi C. (2013). An ACT1 mutation selectively abolishes interleukin-17 responses in humans with chronic mucocutaneous candidiasis. Immunity.

[37] Ling Y., Cypowyj S., Aytekin C., Galicchio M., Camcioglu Y., Nepesov S., Ikinciogullari A., Dogu F., Belkadi A., Levy R. (2015). Inherited IL-17RC deficiency in patients with chronic mucocutaneous candidiasis. J. Exp. Med..

[38] Ferwerda B., Ferwerda G., Plantinga T.S., Willment J.A., van Spriel A.B., Venselaar H., Elbers C.C., Johnson M.D., Cambi A., Huysamen C. (2009). Human dectin-1 deficiency and mucocutaneous fungal infections. N. Engl. J. Med..

[39] Ma C.S., Chew G.Y., Simpson N., Priyadarshi A., Wong M., Grimbacher B., Fulcher D.A., Tangye S.G., Cook M.C. (2008). Deficiency of Th17 cells in hyper IgE syndrome due to mutations in STAT3. J. Exp. Med..

[40] Whibley N., Gaffen S.L. (2014). Brothers in arms: Th17 and Treg responses in Candida albicans immunity. PLoS Pathog..

[41] Manches O., Frleta D., Bhardwaj N. (2014). Dendritic cells in progression and pathology of HIV infection. Trends Immunol..

[42] Chou L.L., Epstein J., Cassol S.A., West D.M., He W., Firth J.D. (2000). Oral mucosal Langerhans’ cells as target, effector and vector in HIV infection. J. Oral Pathol. Med..

[43] Charton-Bain M.C., Terris B., Dauge M.C., Marche C., Walker F., Bouchaud O., Xerri L., Potet F. (1999). Reduced number of Langerhans cells in oesophageal mucosa from AIDS patients. Histopathology.

[44] Rosini S., Caltagirone S., Tallini G., Lattanzio G., Demopoulos R., Piantelli M., Musiani P. (1996). Depletion of stromal and intraepithelial antigen-presenting cells in cervical neoplasia in human immunodeficiency virus infection. Hum. Pathol..

[45] McIlroy D., Autran B., Clauvel J.P., Oksenhendler E., Debre P., Hosmalin A. (1998). Low CD83, but normal MHC class II and costimulatory molecule expression, on spleen dendritic cells from HIV+ patients. AIDS Res. Hum. Retrovir..

[46] Barron M.A., Blyveis N., Palmer B.E., MaWhinney S., Wilson C.C. (2003). Influence of plasma viremia on defects in number and immunophenotype of blood dendritic cell subsets in human immunodeficiency virus 1-infected individuals. J. Infect. Dis..

[47] Donaghy H., Pozniak A., Gazzard B., Qazi N., Gilmour J., Gotch F., Patterson S. (2001). Loss of blood CD11c(+) myeloid and CD11c(−) plasmacytoid dendritic cells in patients with HIV-1 infection correlates with HIV-1 RNA virus load. Blood.

[48] Grassi F., Hosmalin A., McIlroy D., Calvez V., Debre P., Autran B. (1999). Depletion in blood CD11c-positive dendritic cells from HIV-infected patients. AIDS.

[49] Macatonia S.E., Lau R., Patterson S., Pinching A.J., Knight S.C. (1990). Dendritic cell infection, depletion and dysfunction in HIV-infected individuals. Immunology.

[50] Pacanowski J., Kahi S., Baillet M., Lebon P., Deveau C., Goujard C., Meyer L., Oksenhendler E., Sinet M., Hosmalin A. (2001). Reduced blood CD123+ (lymphoid) and CD11c+ (myeloid) dendritic cell numbers in primary HIV-1 infection. Blood.

[51] Pimpinelli N., Borgognoni L., Riccardi R., Ficarra G., Mori M., Gaglioti D., Romagnoli P. (1991). CD36(OKM5)+ dendritic cells in the oral mucosa of HIV− and HIV+ subjects. J. Investig. Dermatol..

[52] Granelli-Piperno A., Golebiowska A., Trumpfheller C., Siegal F.P., Steinman R.M. (2004). HIV-1-infected monocyte-derived dendritic cells do not undergo maturation but can elicit IL-10 production and T cell regulation. Proc. Natl. Acad Sci. USA.

[53] Mann J., Patrick C.N., Cragg M.S., Honeychurch J., Mann D.A., Harris M. (2005). Functional analysis of HIV type 1 Nef reveals a role for PAK2 as a regulator of cell phenotype and function in the murine dendritic cell line, DC2.4. J. Immunol..

[54] Poudrier J., Weng X., Kay D.G., Hanna Z., Jolicoeur P. (2003). The AIDS-like disease of CD4C/human immunodeficiency virus transgenic mice is associated with accumulation of immature CD11bHi dendritic cells. J. Virol..

[55] Schindler M., Wurfl S., Benaroch P., Greenough T.C., Daniels R., Easterbrook P., Brenner M., Munch J., Kirchhoff F. (2003). Down-modulation of mature major histocompatibility complex class II and up-regulation of invariant chain cell surface expression are well-conserved functions of human and simian immunodeficiency virus nef alleles. J. Virol..

[56] Stumptner-Cuvelette P., Morchoisne S., Dugast M., Le Gall S., Raposo G., Schwartz O., Benaroch P. (2001). HIV-1 Nef impairs MHC class II antigen presentation and surface expression. Proc. Natl. Acad Sci. USA.

[57] Messmer D., Jacque J.M., Santisteban C., Bristow C., Han S.Y., Villamide-Herrera L., Mehlhop E., Marx P.A., Steinman R.M., Gettie A. (2002). Endogenously expressed nef uncouples cytokine and chemokine production from membrane phenotypic maturation in dendritic cells. J. Immunol..

[58] Vigerust D.J., Egan B.S., Shepherd V.L. (2005). HIV-1 Nef mediates post-translational down-regulation and redistribution of the mannose receptor. J. Leukoc. Biol..

[59] Segura E., Touzot M., Bohineust A., Cappuccio A., Chiocchia G., Hosmalin A., Dalod M., Soumelis V., Amigorena S. (2013). Human inflammatory dendritic cells induce Th17 cell differentiation. Immunity.

[60] Becattini S., Latorre D., Mele F., Foglierini M., De Gregorio C., Cassotta A., Fernandez B., Kelderman S., Schumacher T.N., Corti D. (2015). T cell immunity. Functional heterogeneity of human memory CD4(+) T cell clones primed by pathogens or vaccines. Science.

[61] Surh C.D., Sprent J. (2008). Homeostasis of naive and memory T cells. Immunity.

[62] van Leeuwen E.M., Sprent J., Surh C.D. (2009). Generation and maintenance of memory CD4(+) T Cells. Curr. Opin. Immunol..

[63] Nagao K., Udey M.C. (2015). Mushrooming insights into skin dendritic cell physiology. Immunity.

[64] Acosta-Rodriguez E.V., Rivino L., Geginat J., Jarrossay D., Gattorno M., Lanzavecchia A., Sallusto F., Napolitani G. (2007). Surface phenotype and antigenic specificity of human interleukin 17-producing T helper memory cells. Nat. Immunol..

[65] Liu Y., Yang B., Zhou M., Li L., Zhou H., Zhang J., Chen H., Wu C. (2009). Memory IL-22-producing CD4+ T cells specific for Candida albicans are present in humans. Eur. J. Immunol..

[66] Elhed A., Unutmaz D. (2010). Th17 cells and HIV infection. Curr. Opin. HIV AIDS.

[67] El Hed A., Khaitan A., Kozhaya L., Manel N., Daskalakis D., Borkowsky W., Valentine F., Littman D.R., Unutmaz D. (2010). Susceptibility of human Th17 cells to human immunodeficiency virus and their perturbation during infection. J. Infect. Dis..

[68] Gosselin A., Monteiro P., Chomont N., Diaz-Griffero F., Said E.A., Fonseca S., Wacleche V., El-Far M., Boulassel M.R., Routy J.P. (2010). Peripheral blood CCR4+CCR6+ and CXCR3+CCR6+CD4+ T cells are highly permissive to HIV-1 infection. J. Immunol..

[69] Prendergast A., Prado J.G., Kang Y.H., Chen F., Riddell L.A., Luzzi G., Goulder P., Klenerman P. (2010). HIV-1 infection is characterized by profound depletion of CD161+ Th17 cells and gradual decline in regulatory T cells. AIDS.

[70] Hu H., Nau M., Ehrenberg P., Chenine A.L., Macedo C., Zhou Y., Daye Z.J., Wei Z., Vahey M., Michael N.L. (2013). Distinct gene-expression profiles associated with the susceptibility of pathogen-specific CD4 T cells to HIV-1 infection. Blood.

[71] Bernier A., Cleret-Buhot A., Zhang Y., Goulet J.P., Monteiro P., Gosselin A., DaFonseca S., Wacleche V.S., Jenabian M.A., Routy J.P. (2013). Transcriptional profiling reveals molecular signatures associated with HIV permissiveness in Th1Th17 cells and identifies peroxisome proliferator-activated receptor gamma as an intrinsic negative regulator of viral replication. Retrovirology.

[72] Brenchley J.M., Paiardini M., Knox K.S., Asher A.I., Cervasi B., Asher T.E., Scheinberg P., Price D.A., Hage C.A., Kholi L.M. (2008). Differential Th17 CD4 T-cell depletion in pathogenic and nonpathogenic lentiviral infections. Blood.

[73] Bixler S.L., Mattapallil J.J. (2013). Loss and dysregulation of Th17 cells during HIV infection. Clin. Dev. Immunol..

[74] Kim C.J., McKinnon L.R., Kovacs C., Kandel G., Huibner S., Chege D., Shahabi K., Benko E., Loutfy M., Ostrowski M. (2013). Mucosal Th17 cell function is altered during HIV infection and is an independent predictor of systemic immune activation. J. Immunol..

[75] Hernandez-Santos N., Gaffen S.L. (2012). Th17 cells in immunity to Candida albicans. Cell Host Microbe.

[76] Weindl G., Wagener J., Schaller M. (2010). Epithelial cells and innate antifungal defense. J. Dent. Res..

[77] Khader S.A., Gaffen S.L., Kolls J.K. (2009). Th17 cells at the crossroads of innate and adaptive immunity against infectious diseases at the mucosa. Mucosal Immunol..

[78] Patel P.K., Erlandsen J.E., Kirkpatrick W.R., Berg D.K., Westbrook S.D., Louden C., Cornell J.E., Thompson G.R., Vallor A.C., Wickes B.L. (2012). The Changing Epidemiology of Oropharyngeal Candidiasis in Patients with HIV/AIDS in the Era of Antiretroviral Therapy. AIDS Res. Treat..

[79] Hanna Z., Kay D.G., Rebai N., Guimond A., Jothy S., Jolicoeur P. (1998). Nef harbors a major determinant of pathogenicity for an AIDS-like disease induced by HIV-1 in transgenic mice. Cell.

[80] Chrobak P., Simard M.C., Bouchard N., Ndolo T.M., Guertin J., Hanna Z., Dave V., Jolicoeur P. (2010). HIV-1 Nef disrupts maturation of CD4+ T cells through CD4/Lck modulation. J. Immunol..

[81] Weng X., Priceputu E., Chrobak P., Poudrier J., Kay D.G., Hanna Z., Mak T.W., Jolicoeur P. (2004). CD4+ T cells from CD4C/HIVNef transgenic mice show enhanced activation *in vivo* with impaired proliferation *in vitro* but are dispensable for the development of a severe AIDS-like organ disease. J. Virol..

[82] Poudrier J., Weng X., Kay D.G., Pare G., Calvo E.L., Hanna Z., Kosco-Vilbois M.H., Jolicoeur P. (2001). The AIDS disease of CD4C/HIV transgenic mice shows impaired germinal centers and autoantibodies and develops in the absence of IFN-gamma and IL-6. Immunity.

[83] Priceputu E., Rodrigue I., Chrobak P., Poudrier J., Mak T.W., Hanna Z., Hu C., Kay D.G., Jolicoeur P. (2005). The Nef-mediated AIDS-like disease of CD4C/human immunodeficiency virus transgenic mice is associated with increased Fas/FasL expression on T cells and T-cell death but is not prevented in Fas-, FasL-, tumor necrosis factor receptor 1-, or interleukin-1beta-converting enzyme-deficient or Bcl2-expressing transgenic mice. J. Virol..

[84] Kay D.G., Yue P., Hanna Z., Jothy S., Tremblay E., Jolicoeur P. (2002). Cardiac disease in transgenic mice expressing human immunodeficiency virus-1 nef in cells of the immune system. Am. J. Pathol..

[85] De Repentigny L., Aumont F., Ripeau J.S., Fiorillo M., Kay D.G., Hanna Z., Jolicoeur P. (2002). Mucosal candidiasis in transgenic mice expressing human immunodeficiency virus type 1. J. Infect. Dis..

[86] Lewandowski D., Marquis M., Aumont F., Lussier-Morin A.C., Raymond M., Senechal S., Hanna Z., Jolicoeur P., de Repentigny L. (2006). Altered CD4+ T cell phenotype and function determine the susceptibility to mucosal candidiasis in transgenic mice expressing HIV-1. J. Immunol..

[87] Hanna Z., Priceputu E., Chrobak P., Hu C., Dugas V., Goupil M., Marquis M., de Repentigny L., Jolicoeur P. (2009). Selective expression of human immunodeficiency virus Nef in specific immune cell populations of transgenic mice is associated with distinct AIDS-like phenotypes. J. Virol..

[88] Goupil M., Cousineau-Cote V., Aumont F., Senechal S., Gaboury L., Hanna Z., Jolicoeur P., de Repentigny L. (2014). Defective IL-17- and IL-22-dependent mucosal host response to Candida albicans determines susceptibility to oral candidiasis in mice expressing the HIV-1 transgene. BMC Immunol..

[89] Huppler A.R., Conti H.R., Hernandez-Santos N., Darville T., Biswas P.S., Gaffen S.L. (2014). Role of neutrophils in IL-17-dependent immunity to mucosal candidiasis. J. Immunol..

[90] Lacasse M., Fortier C., Trudel L., Collet A.J., Deslauriers N. (1990). Experimental oral candidosis in the mouse: Microbiologic and histologic aspects. J. Oral Pathol. Med..

[91] Saunus J.M., Wagner S.A., Matias M.A., Hu Y., Zaini Z.M., Farah C.S. (2010). Early activation of the interleukin-23–17 axis in a murine model of oropharyngeal candidiasis. Mol. Oral Microbiol..

[92] Eversole L.R., Reichart P.A., Ficarra G., Schmidt-Westhausen A., Romagnoli P., Pimpinelli N. (1997). Oral keratinocyte immune responses in HIV-associated candidiasis. Oral Surg. Oral Med. Oral Pathol. Oral Radiol. Endod..

[93] Marquis M., Lewandowski D., Dugas V., Aumont F., Senechal S., Jolicoeur P., Hanna Z., de Repentigny L. (2006). CD8+ T cells but not polymorphonuclear leukocytes are required to limit chronic oral carriage of Candida albicans in transgenic mice expressing human immunodeficiency virus type 1. Infect. Immun..

[94] Goupil M., Trudelle E.B., Dugas V., Racicot-Bergeron C., Aumont F., Senechal S., Hanna Z., Jolicoeur P., de Repentigny L. (2009). Macrophage-mediated responses to Candida albicans in mice expressing the human immunodeficiency virus type 1 transgene. Infect. Immun..

[95] McNulty K.M., Plianrungsi J., Leigh J.E., Mercante D., Fidel P.L. (2005). Characterization of CD8+ T cells and microenvironment in oral lesions of human immunodeficiency virus-infected persons with oropharyngeal candidiasis. Infect. Immun..

[96] Eyerich S., Eyerich K., Pennino D., Carbone T., Nasorri F., Pallotta S., Cianfarani F., Odorisio T., Traidl-Hoffmann C., Behrendt H. (2009). Th22 cells represent a distinct human T cell subset involved in epidermal immunity and remodeling. J. Clin. Investig..

[97] Nurieva R., Yang X.O., Chung Y., Dong C. (2009). Cutting edge: *In vitro* generated Th17 cells maintain their cytokine expression program in normal but not lymphopenic hosts. J. Immunol..

